# Sunitinib Improves Some Clinical Aspects and Reverts DMBA-Induced Hyperplasic Lesions in Hamster Buccal Pouch

**DOI:** 10.1155/2014/859621

**Published:** 2014-02-13

**Authors:** Fernanda Lopes de Souza, Mariana Oliveira, Marianne Brochado Nunes, Lucas Horstmann Serafim, Alan Arrieira Azambuja, Luisa Maria G. de M. Braga, Lisiani Saur, Maria Antonieta Lopes de Souza, Léder Leal Xavier

**Affiliations:** Laboratório de Biologia Celular e Tecidual, Faculdade de Biociências, PUCRS, Avenida Ipiranga 6681, Prédio 12, Sala 104, 90619-900 Porto Alegre, RS, Brazil

## Abstract

Oral squamous cell carcinoma (OSCC) is a public health problem. The hamster buccal pouch model is ideal for analyzing the development of OSCC. This research analysed the effects of sunitinib (tyrosine kinase inhibitor) in precancerous lesions induced by 7,12-dimethylbenz(a)anthracene (DMBA) in this model. Thirty-four male hamsters, divided into six groups: control—C (n = 7), acetone—A (n = 12), carbamide peroxide—CP (n = 5 ), acetone and CP—A+CP (n = 8), 1% DMBA in acetone and CP—DA+CP (n = 6), and 1% DMBA in acetone and CP and 4-week treatment with sunitinib—DA+CP+S (n = 7). The aspects evaluated were anatomopathological features (peribuccal area, paws, nose, and fur), histological sections of the hamster buccal pouches (qualitatively analyzed), epithelium thickness, and the rete ridge density (estimated). Sunitinib was unable to attenuate the decrease in weight gain induced by DMBA; no increase in volume was detected in the pouch and/or ulceration, observed in 43% of the animals in the DA+CP group. DA+CP groups presented a significant increase in rete ridge density compared to the control groups (*P* < 0.01) which was reverted by sunitinib in the DA+CP+S group. Sunitinib seems to have important benefits in early stage carcinogenesis and may be useful in chemoprevention.

## 1. Introduction

Oral squamous cell carcinoma is a global public health problem with about 300,000 new cases diagnosed per year representing 5% of all cancers for men and 2% for women [1], two-thirds of which are from developing countries [2].

Squamous cell carcinoma of the upper aerodigestive tract has a high risk of primary-treatment failure and death. If cured, patients are often disfigured or cannot speak and/or swallow [2]. Some patients will be at risk for malnutrition, infection [3], severe depression, or suicide. Globally, with few exceptions, survival rates have not improved for decades [1, 4–7].

Oral squamous cell carcinoma (OSCC) is caused by DNA mutation, often spontaneous but increased by the exposure to a range of mutagens [8]—one of them being chemical. The changes in the DNA can progress from a normal keratinocyte to a premalignant or a potentially malignant keratinocyte that is characterized by the ability to proliferate in a less-controlled way than normal. The cells become autonomous and cancer results (characterized by invasion through the epithelial basement membrane) [9].

In the initial phase of OSCC, cells may proliferate in a process known as hyperplasia. From hyperplasia, cells can progress to mild dysplasia; then to moderate dysplasia, and later to severe dysplasia; the last phase would be OSCC [1].

Animal tumor models that closely mimic human oral cancers are very important in elucidating the mechanism of neoplastic transformation and so providing leads to effective chemoprevention. The hamster buccal pouch (HBP) carcinogenesis model is the most well-characterized system for analyzing the development of OSCC [5]. The HBP is covered by a thin layer of stratified squamous epithelium that is very similar to the floor of the mouth and the ventral surface of the tongue in humans, which is the most common site of human OSCC [10].

HBP carcinomas are preceded by preneoplastic lesions that are similar to those seen in humans [5]. The early lesions in the HBP look grossly *in vivo* much as they do clinically in humans and the carcinomas are microscopically identical to those seen in humans [11].

Treatment strategies for OSCC are diverse due to the unpredictable behavior of the cancer, local invasion, frequent regional lymph node metastases, and a relative resistance to chemotherapeutic drugs leading to an unpredictable prognosis [12]. The consensus is that the reversal of precancerous lesions or protection from malignant transformation would have a great impact on the prevention and treatment of OSCC [13]. Accordingly, rete ridge density estimation is considered a very important sign when grading oral borderline malignancies [1, 14].

Sunitinib (SU11248) is a selective multitarget small molecule receptor tyrosine kinase inhibitor with antiangiogenic activity that targets the vascular endothelial growth factor (VEGF), platelet-derived growth factor (PDGF), KIT, and FLT3 receptor tyrosine kinases. Through the same pathway, it can exhibit direct antitumor activity against tumor cells that depend upon this signaling to proliferate/survive [15, 16]. In mouse xenograft models, sunitinib exhibits a wide and potent antitumor activity causing regression, growth arrest, or substantially reducing growth of various tumor cell lines [15]. Sunitinib interferes in some alterations that in cell physiology collectively dictate malignant growth, such as growth signal autonomy of tumoral cells and sustained angiogenesis [17].

Sunitinib is approved by the United States Food and Drug Administration for the treatment of imatinib-resistant/imatinib-intolerant gastrointestinal stromal tumour (GIST), advanced renal cell carcinoma, and pancreatic neuroendocrine tumours [16] and is being tested for use against other solid tumours [18].

Thus, the goals of this study were to evaluate qualitatively and quantitatively the effect of sunitinib in precancerous lesions induced by 7,12-dimethylbenz(a)anthracene (DMBA) in the hamster buccal pouch. Additionally, important clinical aspects of these animals were described such as weight gain and the clinical aspects of the peribuccal area, paws, nose, and fur.

## 2. Materials and Methods

### 2.1. Animals

The experiment was performed using 34 five-week-old male Syrian golden hamsters (*Mesocricetus auratus*), obtained from a breeding colony from Universidade Federal de Pelotas, Rio Grande do Sul, Brazil. Animals were housed in standard boxes (two per cage) under standard laboratory conditions (temperature 20°C ± 1°C, 12 h light/dark cycle, with standard chow and water *ad libitum*). All experiments were performed in accordance with the NIH Guide for Care and Use of Laboratory Animals (USA) and the Brazilian Laws for animal care and ethical use of animals [19]. The study was approved and the number of animals to be used was determined by the Ethics Committee of the *Pontificia Universidade Catolica do Rio Grande do Sul* (PUCRS) (Protocol no. CEUA-PUCRS 10/00171); all efforts were made to minimize animal suffering.

### 2.2. Experimental Design

The animals were randomly divided into six groups: control (C) (*n* = 7), acetone (A) (*n* = 12), carbamide Peroxide (CP) (*n* = 5), acetone + carbamide peroxide (A+CP) (*n* = 8), DMBA in acetone + carbamide Peroxide (DA+CP) (*n* = 6), and DMBA in acetone + carbamide peroxide + sunitinib (DA+CP+S) (*n* = 7).

The experiment was divided in two phases. The first 55 days were the induction phase and the second phase was the treatment (sunitinib) phase that lasted 4 weeks.

During the 55 days of induction, in group C, no product was applied to the right buccal pouch. In A group, acetone was applied using a number 4 marten's fur brush, 3 times per week, in the right buccal pouch. In the CP group, a 10% carbamide peroxide gel (Opalescence 10%, Ultradent Products, Inc. South Jordan, UT) was applied 2 times per week in the right buccal pouches of the hamsters. In group A+CP, acetone was applied 3 times per week and in the same buccal pouch carbamide peroxide was applied 2 times per week. In groups DA+CP and DA+CP+S, 1% DMBA diluted in acetone 3 times per week were applied and carbamide peroxide 2 times per week on the right buccal pouch throughout the induction protocol. The only difference was that DA+CP+S was the only group treated with sunitinib (treatment phase), while all the other groups were left untreated during the last 4 weeks of the experiment.

In groups A, CP, and A+CP, only part of the induction protocol was used to determine any interference of these agents in the epithelial hyperplasia-inducing protocol.

### 2.3. Precancerous Induction

The precancerous induction was performed using a solution of DMBA (1%) (Sigma Chemicals, Co., USA) diluted in acetone.

In the animals in groups DA+CP and DA+CP+S, 1% DMBA diluted in acetone was applied in the fundus of the right buccal pouch, using a number 4 marten's fur brush, 3 times per week. The amount of DMBA delivered to each animal was quite uniform using the “wiped-brush” technique [20].

Two times a week, in the same buccal pouch where the DMBA was applied, a predetermined amount of 10% carbamide peroxide was applied using the applying tip that comes with the product. Carbamide peroxide is an important coadjuvant for carcinogenesis in this model [21, 22].

### 2.4. Sunitinib Treatment

The drug treatment protocol and the dose chosen were similar to those used in previous studies made with rodents using sunitinib as cancer treatment. The animals received 40 mg of sunitinib/kg/day by oral gavage during 4 weeks [15, 23, 24]; the drug was diluted in distilled water immediately before use.

At the end of the induction period (55 days), all animals were weighed to determine the amount of sunitinib that should be used. After that, the animals were weighed weekly and the dilution was recalculated and adjusted every week.

### 2.5. Weight

The animals were weighed at the beginning and the end of the induction period and weekly during the treatment phase with sunitinib.

### 2.6. Histological Procedures

At the end of experimental period, all animals were euthanized by administration of a lethal dose of sodium thiopental (50 mg/kg, i.p.; Cristália, Brazil) and transcardially perfused using a peristaltic pump (Milan, Brazil) with 300 mL of saline solution, followed by 400 mL of 4% paraformaldehyde (Reagen, Brazil) in 0.1 M phosphate buffer (PB, pH 7.4) at room temperature. Both buccal pouches of all animals were dissected, removed, and postfixed in the same solution used for fixation. The pouches were divided into equal parts, measuring 1 cm each, starting at the opening of the pouch to the fundus, included in paraffin wax, sectioned at equidistant intervals using a rotary microtome (10 *μ*m) (Leica RM-2255, Germany), and stained with hematoxylin and eosin.

## 3. Morphometrical Analysis

The digitized images of the buccal pouch sections were obtained using an Olympus BX 50 microscope (4x and 20x) coupled to a video camera (Leica DC 300F) interfaced by Leica Image 50 (IM50) software. The images obtained were measured using Image Pro Plus Software (Image Pro-Plus 6.1; Media Cybernetics, Silver Spring, MD, USA) and at least three images of each pouch per animal were analyzed. The epithelium thickness and the rete ridge density were measured.

The epithelium thickness was estimated by measuring the distance between the most superficial layer of the epithelium and the basal membrane. Three equidistant sites were measured in every image.

To estimate the rete ridge density, an adaptation of the protocol initially described by Klein-Szanto and Schroeder [25] was performed. The length of the most superficial epithelium layer was measured and the number of rete ridges in that image was counted [26]. The number of rete ridges was divided by the length of the epithelium and the result was considered the rete ridge density, expressed by the following unit: number of rete ridges/mm. Both measurements were performed by two blinded, prestandardized investigators.

## 4. Statistical Analysis

The statistical analysis of weight and morphometric data was performed using one-way ANOVA followed by the Tukey test (*P* < 0.05). All statistical procedures were performed using the SPSS 15.0 software (Statistical Package for the Social Sciences, Chicago, IL, USA).

## 5. Results

### 5.1. Weight Gain

**Figure 1:**
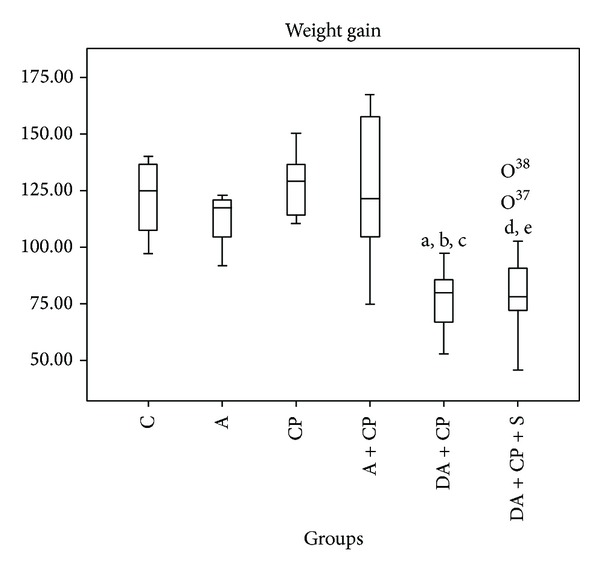
Weight gain. Weight gained during the experimental period (83 days), expressed in grams, where a = DA+CP *≠* A, b = DA+CP *≠* CP, c = DA+CP *≠* A+CP (*P* < 0.01), d = DA+CP+S *≠* CP (*P* < 0.001), and e = DA+CP+S *≠* A+CP (*P* < 0.01).

In our study, we observed that, during the 83 experimental days, there was a statistically significant lower weight gain in the DA+CP and DA+CP+S groups when compared to the control groups (Figure 1).

### 5.2. Clinical Aspects

As soon as the experiment began, most of the manipulated animals stopped using the right buccal pouch for food storage, preferring the left one. During the induction phase, the animals in the DA+CP and DA+CP+S groups presented similar clinical alterations described as follows: in a first stage, the peribuccal area of the pouch presented inflammatory lesions with small ulceration and suppuration. Soon after, the area started losing fur; the ulceration was still present but suppuration lessened.

**Figure 2:**
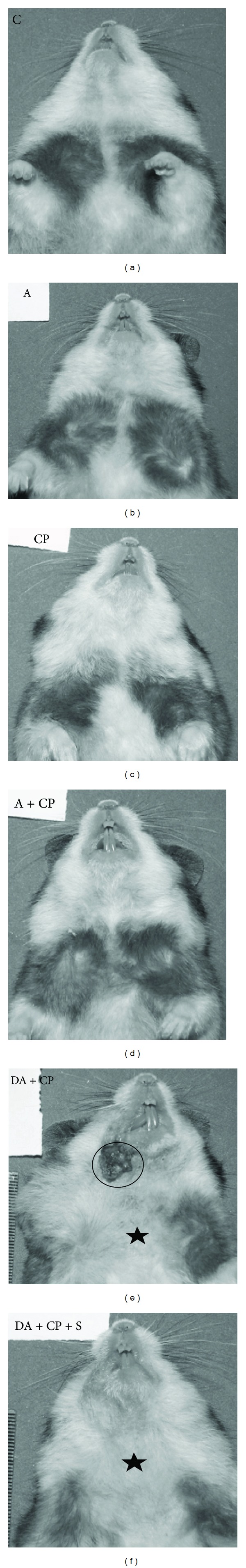
Digitized images showing some of the clinical aspects of the animals at the end of the experimental period; note the area of fur loss (star) and fistula (inside the circle) on the animal from the DA+CP group. C: control group, A: acetone group, CP: carbamide peroxide group, A+CP: acetone and carbamide peroxide group, DA+CP: 1% DMBA in acetone and carbamide peroxide group, DA+CP+S: 1% DMBA in acetone and carbamide peroxide, and treatment with sunitinib group.

At the end of the experimental period, we observed that sunitinib had inhibited tumoral growth and/or the presence of ulceration, as were observed in three animals in the DA+CP group (43%) (Figure 2).

The animals treated with sunitinib presented the signs typical of patients undergoing treatment with sunitinib [16], such as cold paws with edema and a yellowish color easily seen on the nose and paws; the urine was also intensely yellow, possibly caused by the drug color.

### 5.3. Qualitative Histological Analysis

In the histological examination, the keratin, epithelial, and connective tissue layers were examined. A histological description of every group was made and measurements of the epithelium were registered.

In all groups, most of the slices examined presented a very thin keratin layer. In the control groups (C, A, CP, and A+CP) and animals treated with sunitinib (DA+CP+S), the epithelium-connective tissue relation was a straight line. Epithelium did not increase in thickness and the connective tissue did not show alteration. The main feature that caught our attention in the DA+CP group was the epithelium-connective tissue relation, with prominent rete ridges and several connective tissue papillae (Figure 4).

### 5.4. Precancerous Lesions

Two parameters were used to determine the presence of precancerous lesions: the epithelium thickness and the rete ridge density.

### 5.5. Epithelium Thickness

**Figure 3:**
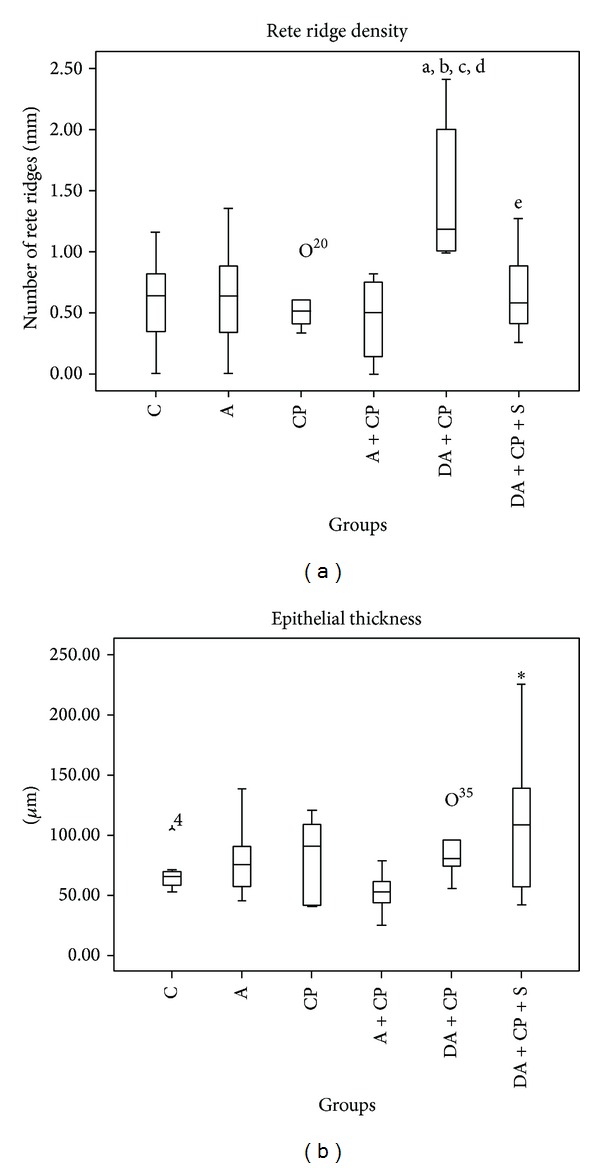
(a) Epithelial thickness, expressed in µm. (b) rete ridge density, expressed in number of rete ridges/mm, where a is DA+CP *≠* C (*P* < 0.01), b is DA+CP *≠* A (*P* < 0.01), c is DA+CP *≠* CP (*P* < 0.01), d is DA+CP *≠* A+CP (*P* < 0.001), and e is DA+CP+S *≠* DA+CP (*P < 0.05).

In relation to the epithelium thickness, a significant increase was found in the DA+CP+S group when compared to the A+CP group (*P* < 0.05) (Figure 3(a)).

### 5.6. Rete Ridge Density

In the DA+CP group, there was a significant increase in rete ridge density when compared to the control groups: group C (*P* < 0.01), group A (*P* < 0.01), group CP (*P* < 0.01), and group A+CP (*P* < 0.001) (Figure 3(b)).

**Figure 4:**
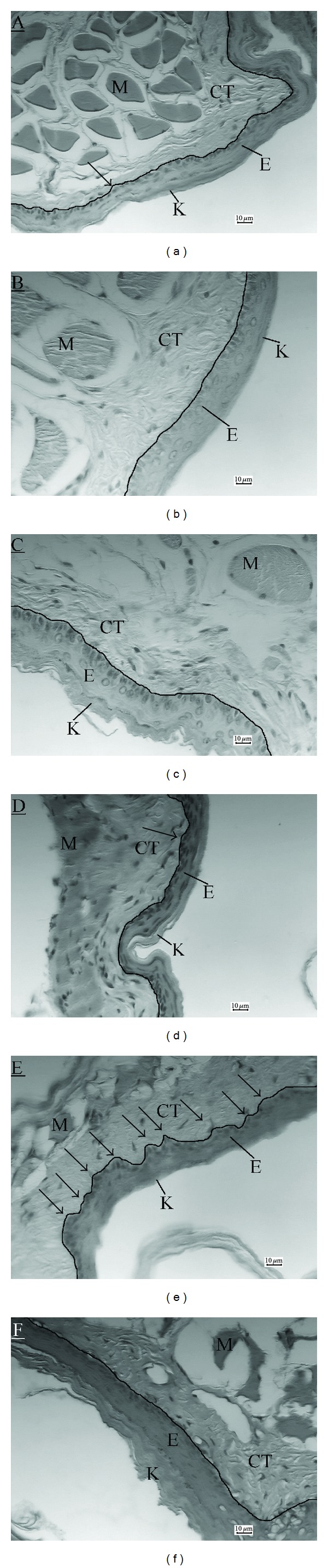
Digitized images of histological sections of the right hamster buccal pouch, stained with hematoxilin and eosin. Note an increased number of rete ridges in the DA+CP group that was reverted by sunitinib in the DA+CP+S group. (a) control group, (b) acetone group in which, acetone was applied 3 times/week, (c) 10% carbamide peroxide which was applied 2 times/week, (d) Acetone + carbamide Peroxide group, (e) DA+CP group, and (f) DA+CP+S group. M: striated muscle, CT: connective tissue, E: epithelium, K: keratin, and arrow: rete ridge.

Comparing precancerous lesion induction without (DA+CP) and with sunitinib treatment, a significant decrease in rete ridge density was found in the group exposed to DMBA and treated with sunitinib (DA+CP+S) (*P* < 0.05) (Figures 3(b) and 4).

## 6. Discussion

In our study, we observed that DMBA treatment induced a significant reduction in weight gain in the DA+CP and DA+CP+S groups (Figure 1). Different hypotheses could be formulated to elucidate this finding; one possible explanation is that one of the side effects of treatment with sunitinib is weight loss, as described by Bagi et al. [18] when studying the effects of an eight-day sunitinib treatment for hepatocellular carcinoma in mouse. In this study, animals with cancer gained less than 5% body weight, while animals with cancer and treated with sunitinib lost about 5% of their body weight. Our results are more promising than Bagi's results since we observed a slower weight gain in the DA+CP and DA+CP+S groups rather than weight loss. These weight differences between the results found in the present study and those reported by Bagi are probably due to the different protocols used.

To produce the precancerous lesions, we used a protocol that is very similar to other well-described protocols to induce OSCC in HBP [13, 21, 27, 28]. The switch to an angiogenic phenotype in hamster buccal pouch carcinogenic model is a less discrete event that occurs between the third and fifth week after the beginning of the induction protocol [11]. Somewhere between 6 weeks and 10 weeks [13, 27, 28] following the initiation of the protocol, dysplastic lesions are found [1].

Our 55-day protocol using DMBA at 1% was enough to induce the angiogenic switch intended and avoid all the stress caused by the presence of OSCC in the animal's buccal pouches.

In this protocol, carbamide peroxide was applied as a promoter. When used as clinically indicated, if it comes in contact with the oral mucosa, 10% carbamide peroxide is capable of causing morphological changes in the gingival epithelium, as well as increasing the proliferation rate of epithelial cells [22]. This was the action intended in our study. So DMBA was employed to stimulate the presence of mutant cells and carbamide peroxide to increase the proliferation rate of the cells.

We also tested if carbamide peroxide alone could promote significant changes in the HBP epithelium (group CP). Our findings showed that only the association (DMBA + carbamide peroxide) was able to change the epithelial morphology, generating precursor lesions (Figure 4).

In the first parameter analyzed: epithelial thickness, a significant difference was found when comparing DA+CP+S with A+CP (Figure 3(a)). At present, we may suggest that the increased epithelial thickness found in the A+CP group could be a response to the combination of irritating agents used, and a thickness increase without morphological alteration of the epithelial layers was a protective response.

Another goal of our study was to evaluate the effects of sunitinib in cells with cancer-associated genetic alterations, but with no invasive growth [29].

It can be clearly observed in the DA+CP group that there is an alteration in the basal layer of the epithelium with a proliferative aspect without connective tissue invasion, that could be classified as a hyperplasia or mild dysplasia [1] (Figure 4).

Knowing that sunitinib targets the angiogenic activity in tumors [15, 18, 23, 24] and that OSCC needs angiogenesis to proliferate and invade connective tissue, we wondered if sunitinib would interfere in the initial process (hyperplasia and dysplasia) of OSCC.

The HBP epithelium consists of a thin, regular, keratinized stratified squamous epithelium, the epithelium-connective tissue junction being relatively flat, and rete ridges rarely observed [30, 31]. Next to the connective tissue, there is a striated muscle layer mixed with soft connective tissue [21]. This histological characteristic is found in all the control groups (Figure 4).

In the second parameter analyzed, an increase in rete ridge density in the DA+CP group was observed (Figure 3(b)). In the group exposed to DMBA and treated with sunitinib (DA+CP+S), sunitinib can be seen to have reverted the increase in rete ridge density induced by DMBA (*P* < 0.05) (Figure 3(b)). Rete ridges are adaptive structures that enlarge the epithelium-connective tissue interface in order to achieve a better anchorage for the epithelium and provide a larger exchange surface for nutritional purposes [32, 33]. They are a key feature when grading oral borderline malignancies [14].

Oliveira et al. [21] showed that, even in the initial phase of tumor induction protocol (55 days and 70 days), a rich new vascular network was formed. This network is necessary to allow tumor growth. Sunitinib would act in this angiogenic area, reducing the number of new vessels or avoiding their formation, making it more difficult for the proliferating cells to find a nutritional source. We believe that would be the reason why the rete ridge density was lower in the group treated with sunitinib (DA+CP+S) (Figure 4), since sunitinib targets several molecules important for angiogenesis [23].

## 7. Conclusion

In conclusion, to our knowledge, this study is the first to demonstrate some beneficial effects of sunitinib treatment in an animal model of oral precancerous lesions. The main benefits found were reduction of the precancerous lesion growth and ulceration incidences and reduction, to a normal state, of the rete ridge density.

More research should be done, but our study suggests that sunitinib treatment should be considered as one possible alternative for the treatment of oral cancer.
